# The Role of Oxytocin in Antisocial Personality Disorders: A Systematic Review of the Literature

**DOI:** 10.3389/fpsyt.2019.00076

**Published:** 2019-02-27

**Authors:** Trevor Gedeon, Joanne Parry, Birgit Völlm

**Affiliations:** ^1^NHSHSW Rampton High Secure Hospital, Retford, United Kingdom; ^2^Wathwood Hospital, Rotherham, United Kingdom; ^3^Klinik und Poliklinik für Forensische Psychiatrie, Universitätsmedizin Rostock, Rostock, Germany

**Keywords:** antisocial, ASPD, dissocial, antisocial personality disorder, personality, personality disorder, oxytocin

## Abstract

**Background and aims:** Antisocial personality disorder is an enduring mental disorder associated with significant disease burden and treatment difficulties. This is apparent within forensic populations. There is growing evidence to suggest that treatment with oxytocin could have some benefit in treating a range of psychiatric disorders. There are no reviews studying the use of oxytocin for patients with ASPD. We aim to present the first literature review on the use of oxytocin in patients with ASPD.

**Method:** We searched relevant databases for original research on effect of oxytocin upon persons with a diagnosis of ASPD or healthy participants with symptoms seen in ASPD. Studies were included if they included healthy participants that evaluated the effect of oxytocin on symptoms relevant to ASPD, including empathy, inhibitory control, compliance, conformity, aggression, violence, and moral responsibility.

**Results:** Thirty-six studies were included. There were a range of study designs, including randomized controlled trials, double blinded, single blinded, and unblinded controlled trials. The sample sizes in studies ranged from 20 to 259 participants. Studies looked at participants with a diagnosis of ASPD and participants with symptoms relevant to ASPD, including empathy, inhibitory control, compliance, conformity, aggression, violence, and moral responsibility. Oxytocin was found to demonstrate diversified effects, in most cases being associated with socially positive or non-criminogenic behaviors. However, some studies found opposite, and non-desirable, effects, e.g., an increase in violent inclinations to partners. The two studies looking at participants with ASPD had a number of limitations and had conflicting results on the impact that OT has on aggression in ASPD.

**Conclusions:** This is the first systematic literature review exploring the potential use of oxytocin in managing ASPD and the symptoms of ASPD. It is apparent that there is a body of evidence addressing related symptoms in healthy individuals. There were diversified effects with oxytocin showing some benefits in promoting positive effects on symptoms of ASPD, but there were also studies showing non-desirable effects. It is difficult to draw any direct inferences from healthy control studies. Further high quality large sample studies are required to explore the effects of oxytocin in those with ASPD

## Introduction

Personality disorders are a group of enduring mental disorders characterized by maladaptive patterns of behavior, cognition, and inner experience. These traits are relatively stable across time and situations (1).

Personality disorders are relatively common mental disorders. An epidemiological study of the prevalence of personality disorders in a random sample of 626 British households found that the prevalence of any personality disorder was 4.4% (2). In treatment settings, both primary care and general psychiatric settings, the prevalence of personality disorders is significantly higher. In a sample of 859 psychiatric outpatients in America 31.7% had a diagnosis of a personality disorder (3). A systematic literature review identified that the prevalence of personality disorder in community secondary psychiatric care in Europe was between 40 and 92% (4).

Personality disorders are a source of distress and suffering for patients and those around them. People with personality disorders have been found to use mental health services more than those with major depressive disorders (5). In addition, patients with personality disorders have been found to have greater social dysfunction than those with many other mental disorders (6). In addition, the costs of personality disorders are high. An economic study of patients with personality disorders in the Netherlands found that treatment-seeking patients with personality disorders pose a high economic burden on society at a mean cost of €11,126 per year (7). A study in England found that the cost to the NHS and prison service of those with a personality disorder before treatment was £13,966 per year (8).

Individuals with antisocial personality disorder (ASPD) are of particular concern as they may cause harm to others. Symptoms include a failure to conform to social norms, repeated deceitfulness, impulsivity, irritability, and aggression, consistent irresponsibility, disregard for their own safety or the safety of others and a lack of remorse (1). The prevalence of ASPD in the community has been estimated at 0.6% (2). In a study of psychiatric inpatients aged between 18 and 37 in the UK, the prevalence of ASPD was 14% (9). An Office of National Statistics (ONS) survey of prisoners in England and Wales found a prevalence of any personality disorder of 78% for male remand, 64% for male sentenced, and 50% for female prisoners, the majority of which accounted for by ASPD (10). A review of the international literature found a prevalence of ASPD within custodial settings of 47% (11).

There are a number of theories on the etiology of ASPD. These include genetic, neurobiological and environmental models (12). More recently studies have also looked at specific neurobiological factors, such as the role of the hormone oxytocin and polymorphisms in the oxytocin receptor gene (13).

Oxytocin is a neuropeptide produced in the supraoptic and paraventricular nuclei of the hypothalamus. It is involved in a wide range of bodily reactions via interactions with sex organs and hormones and the Hypothalamic Pituitary Axis (HPA). As such Oxytocin is involved in a range of physiological processes including sexual activity, pregnancy, lactation, social bonding, pain regulation, and maternal behavior (14, 15). Oxytocin is also central to various aspects of human behavior such as social cognition, affectivity, stress response, affiliation, and prosocial behavior (15, 16). Manipulation of oxytocin levels has been shown to alter social cognition in healthy individuals, e.g., increase social interaction, empathy and trust, and reduce stress (17). In a double blind placebo controlled crossover trial of intranasal oxytocin, those given oxytocin performed better on a fear recognition task compared with those given placebo (18); they also demonstrated more positive communication and had lower salivary cortisol levels in response to conflict (19).

Due to these attributes oxytocin and its potential clinical applications have been studied in relation to a number of mental disorders, including autistic spectrum disorders, schizophrenia, depression, and anxiety. In a randomized controlled double blind placebo controlled trial of 33 adult men with high functioning autism subjects were given intranasal oxytocin and their performance on a social psychological task was assessed. The authors concluded that oxytocin has a beneficial effect on the socio-communicational deficits in autism, as patients were able to make non-verbal judgments more quickly compared with those in the placebo condition (20).

A double-blind placebo-controlled crossover study of 21 patients with schizophrenia found an improvement in emotional facial recognition following administration of intranasal oxytocin (21). In another randomized control trial, 20 patients with schizophrenia demonstrated a significant reduction in Positive and Negative Syndrome Scale (PANNS) scores and an improvement in several social cognition measures (22).

The potential application in personality disorders of oxytocin have not yet been explored. The current guidance on the treatment of ASPD highlights that the evidence base for both pharmacological and psychological interventions is limited and recommends that “Pharmacological interventions should not be routinely used for the treatment of antisocial personality disorder or associated behaviors of aggression, anger and impulsivity” (23). A Cochrane systematic review on the use of psychological and pharmacological interventions in ASPD also highlight the limited evidence base and insufficient evidence to support either pharmacological or psychological therapies (24).

As noted above, individuals with ASPD display a number of symptoms, which, based on the evidence in healthy controls as well as individuals with other disorders, may be positively affected by oxytocin. These symptoms include lack of empathy, one of the diagnostic features for a diagnosis of ASPD. Whilst deficits in empathy can be present in a number of psychiatric disorders, including other personality disorders, psychotic disorders, and autistic spectrum disorders, these deficits do not form part of the diagnostic criteria in any other condition. Other potential target symptoms include lack of conformity and compliance and lack of moral reasoning (1, 25).

The aim of this review is therefore to provide an overview of the literature on the use of oxytocin in ASPD as well as targeting key symptoms of the disorder.

## Method

In conducting this review, we have followed the PRISMA guidelines for reporting systematic reviews (26).

### Search Strategy

We undertook a systematic literature search of publications up until March 2018. The search was undertaken with the assistance of an information specialist of the Nottinghamshire Healthcare NHS Foundation Trust Library Service and included the electronic databases MEDLINE, EMBASE, PsycINFO CINAHL, Cochrane Library, ASSIA, Sociological Abstracts, BIOSIS, Web of science. In addition, the EU Clinical Trials Register (www.clinicaltrialsregister.eu), the clinical trials register of the U.S National Institute of Health (www.clinicaltrials.gov) and dissertation abstracts were searched for any ongoing trials relevant to our review. The search terms used related to the DSM V classification for ASPD and various terms relating to oxytocin. The full search strategy is included in Appendix 1 in Supplementary Material.

Results of the searches were reviewed independently by authors JP and TG for suitability for inclusion in the review against the criteria set out below. This was initially undertaken through inspection of titles and abstracts. A second review appraising the full papers was then undertaken as required. In the event of a difference of opinion over a paper's suitability for inclusion a third author (BV) was consulted. Additionally, authors JP and TG searched reference lists from both included and excluded studies for further suitable papers for inclusion.

### In- and Ex-clusion Criteria

Studies of any type of design were included if they met the following criteria:

- Original research
- Studies where oxytocin was administered as the primary intervention
- Studies where participants had a diagnosis of ASPD
- Studies with healthy participants that evaluated the effect of oxytocin on symptoms relevant to ASPD, including empathy, inhibitory control, compliance, conformity, aggression, violence, and moral responsibility
- Human participants over the age of 18
- Male and female participants
- All study sizes
- Studies in all languages and from all countries
- Studies were excluded if participants had a comorbid major mental illness due to the potential for the confounding impact that these disorders may have upon any treatment effect. This was defined as having any presence of any comorbid mental disorder, specifically; organic, developmental, addictive, neurotic, affective or psychotic disorders as categorized by DSM V.

## Results

### Search Results

**Figure 1:**
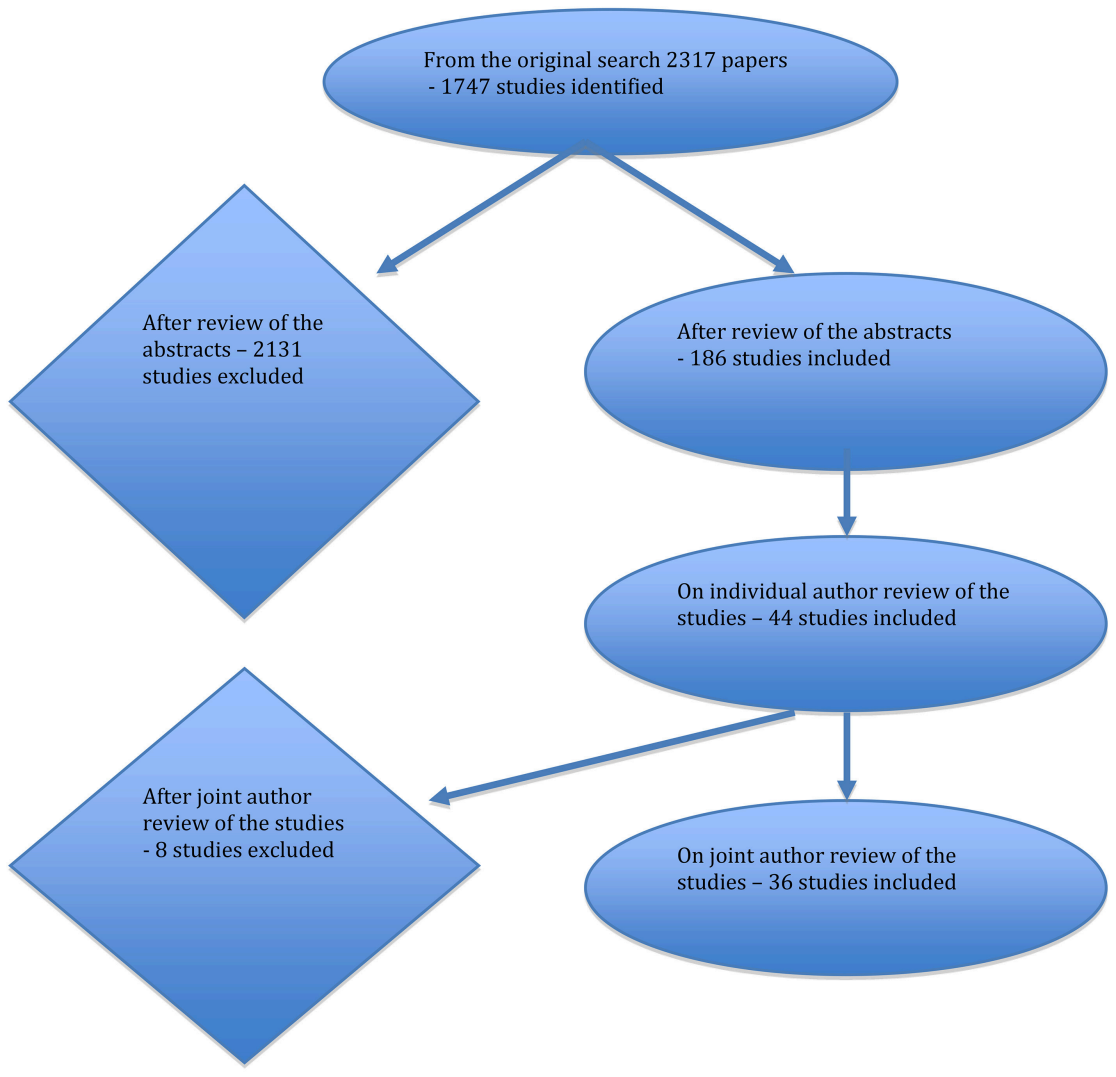
Flow of literature search results.

The initial searches returned 2,317 potentially relevant titles. Following inspection of titles and abstracts 186 full text papers were obtained and assessed against our inclusion criteria of which 36 were deemed relevant and were included in this review. A flow chart of search results is set out in Figure 1. Details of the studies are shown in Table 1.

**Table 1:** Summary table of included studies.

**References**	**Study type**	**Participants**	**Intervention**	**Outcome measures**	**Findings**
**STUDIES IN PARTICIPANTS WITH ASPD**					
Timmerman et al. (27)	Randomized, controlled, double blind, placebo crossover trial	22 adults with ASPD (14 males 8 females) and 29 healthy controls (11 females, 18 males)	IN OT (24 IU) or placebo	Participants were shown images of faces showing various emotions and assessed on their ability to accurately identify the emotion displayed and the time delay for this	Relative deficits in the ASPD group recognizing fearful and happy faces were no longer observable under OT
Alcorn et al. (28)	Single blind placebo controlled trial	6 male participants with ASPD	IN OT (12, 24, 48 IU) or placebo	Performance on a validated laboratory task of human aggression (point subtraction aggression paradigm—PSAP)	There were few differences between those on placebo and those with OT in performance on the PSAP
**STUDIES IN HEALTHY PARTICIPANTS**					
**Studies in empathy**					
Human et al. (29)	Randomized, controlled, double blind placebo controlled trial	116 healthy participants	IN OT (40 IU) or placebo	Following being helped on a task participants affect and social perceptions were rated	In the context of being helped by a stranger oxytocin fostered more positive affective and social responses
Hubble et al. (30)	Randomized, controlled, double blind, placebo within subject trial	40 healthy males	IN OT (24 IU) or placebo	Participants completed questionnaires which provided empathy scores after being shown emotion eliciting video clips. In addition the eye tracking of the participants was assessed	OT was associated with an increase in time spent fixating upon the eye region of the protagonists face across emotions. OT selectively enhanced self-reported affective empathy for fear but not other emotions. There was no positive relationship between eye gaze patterns and affective empathy
Hecht et al. (31)	Randomized, double blind, placebo controlled trial	28 healthy females	IN OT (24 IU) or placebo	Neural responses on fMRI to participants being shown animations of geometric shapes depicting social interactions	Lower social processing at baseline at baseline predicts a more positive response to OT
Li et al. (32)	Randomized, controlled, double blind, placebo within subject trial	30 healthy fathers of 1-2 year old children	IN OT (24 IU) or IN AVP (20 IU) followed by placebo	Brain function was measured with fMRI with the participants viewing images of their children, unknown children and unknown adults as they listened to a crying stimulus	OT but not AVP increased the participants responses to images of their own children
Luo et al. (33)	Randomized, controlled, double blind placebo controlled trial.	86 healthy participants	IN OT (24 IU) or placebo	Brain function was measured with fMRI with the participants viewing a range of images of emotional faces	Oxytocin produces sex dependent effects even at the early stages of social processing
Strang et al. (34)	Randomized, controlled, double blind placebo controlled trial	132 healthy male participants	IN OT (24 IU) or placebo	Performance on a social discounting task as a measure of generosity	The effect of oxytocin on generous behavior is modulated by trait empathy. In those who were administered oxytocin there was a positive correlation between trait empathy and their generosity
Hi et al. (35)	Randomized double bind cross over study	41 healthy males	IN OT (24 IU) or placebo	Performance on the “HelPun” task to examine the altruistic decision making of participants to help or punish others in the task. fMRI scanning before and after OT or placebo administration	In the OT group there was a trend to accelerate altruistic decision making. In the OT group there was enhanced activity in the left temporo parietal junction during observation of others being helped by the computer. These results indicated that OT enhances prosocial revelant perception by increasing theory of mind related neural activities
Korb et al. (36)	Randomized, double blind, placebo controlled, between subject trial	60 healthy male participants	IN OT 24 IU or placebo	Performance on the Offset and Intensity facial mimicry tests as assessed compared with baseline facial EMG	Facial mimicry was increased in the OT group. These effects were strongest for angry infant faces
Palgi et al. (37)	Double blind, within subject placebo randomized controlled trial	30 male and female participants	Single dose IN OT (24 IU) or placebo	Participants listened to recording of mixed gender protagonists describing distressing emotional conflicts and were then asked to provide compassionate advice	In male and female participants OT enhanced compassion toward females but not males
Perry et al. (38)	Randomized double blind placebo controlled trial	54 male participants	Single dose IN OT (24 IU) or placebo	Online questionnaire assessing empathy Experiment involved participants indicating their preferred interpersonal distance	Among highly empathetic individuals OT promoted choice of closer interpersonal distances while the opposite effect was found in individuals with low empathetic traits
Gallup and Church (39)	Randomized double blind placebo control trial	60 male healthy participants	Single dose IN OT (30 IU) or placebo	Exposure to a continuous yawning video	OT did not increase contagious yawning but modulated expression indicative of awareness of social stigma associated with this behavior
Abu-Akel et al. (40)	Double blind placebo controlled crossover trial	29 male and female participants	Single dose IN OT (24 IU) or placebo	Self-perspective empathy vs. other perspective empathy in painful and non-painful situations	OT but not placebo, increased other perspective empathy
Cardoso et al. (41)	Randomized double blind placebo control trial	82 male and female participants	Single dose IN OT (24IU) or placebo	Perceiving and understanding emotion components of MSCEIT.=	OT led to participants rating emotion in facial stimuli as expressing greater emotional intensity than those on placebo. Accurate identification of type of emotion in faces impaired in OT group.=
Fischer-Shofty et al. (42)	Double blind placebo controlled crossover trial	62 male and female participants	Single dose IN OT (24 IU) or placebo	Interpersonal perception task	OT improved accurate perception of social interactions. OT also had sex specific impacts—improved kinship recognition within women but not men; performance of males was only improved on competition recognition
**Studies in inhibitory control**					
Hirosawa et al. (43)	Single blind placebo controlled crossover study	20 male participants	IN OT (24 IU) or placebo	Paradigm 1: Facial cognition. Paradigm 2: attentional-inhibitory control using a modification of the speeded flanker task.	No significant behavioral effects of OT. However, the enhancement of attentional inhibitory control after OT administration significantly correlated to the positively valenced effects of the interpretation of uncertain facial cognition
Ma et al. (44)	Double blind placebo controlled between subject design	150 Male participants	IN OT (24 IU) or placebo	Task of in-group favoritism where cognitive processing was experimentally manipulated. Participants were also assessed for intuition or reflection in daily life	OT increased in-group favoritism in intuitive participants but decreased it in those who rely on reflective style
**Studies in compliance and conformity**					
Aydogan et al. (45)	Randomized, double blind, placebo controlled trial	120 healthy males	IN OT (24 IU) or placebo	Performance on a competitive and noncompetitive coin tossing task, where participants would self-report in order to win a monetary prize. This task was to measure conformity to the widely accepted norm of honesty under the pressure of competition in the OT group compared with the placebo group	Conformity was enhanced by oxytocin and this enhancement had a detrimental effect on honesty in a competitive environment but not in a noncompetitive environment
Gross and De Dreu (46)	Randomized, controlled, double blind, placebo within subject trial	139 healthy participants	IN OT (24 IU) or placebo	Performance on a test of conformity to instructions. In the test, participants had a binary choice and were given an arbitrary rule that would mean that they would receive a lesser financial benefit	Under oxytocin participants violated the rule more often. This was most apparent in individuals who had a high need for structure
Lambert et al. (47)	Randomized, double blind, placebo controlled trial	30 healthy females	IN OT (24 IU) or placebo	Participants performance on 2 social dilemma games was measured whilst participants were also shown social cues in the form of pictures of neutral or angry faces. During the tasks, an fMRI scan was conducted	OT significantly increased the activation of the nucleus accumbens during an assurance game that rewards mutual cooperation. OT significantly attenuates the amygdala
Ten Velden et al. (48)	Randomized, controlled, double blind, placebo within subject trial	65 healthy males and 129 healthy female participants	IN OT (24 IU) or placebo	Participants were placed in groups and given tasks to test the levels of cooperation with the in-group. The task involved the group deciding to make an within group contribution or a between group contribution, Prior to the decision to contribute participants undertook a Stroop Interference task that was either cognitively taxing or not	Participants receiving placebo contributed more to the within group when they were cognitively taxed. The OT group contributed to the within group regardless of cognitive taxation
Hertz et al. (49)	Randomized, double blind, placebo controlled trial	90 healthy male participants	IN OT (40 IU) or placebo	Performance in paired dyads on a visual search task	Compared to the placebo group there was a greater collective benefit over time in the OT group. In the OT group, the more competent member of each dyad was less likely to change his mind during disagreements
Edelson et al. (50)	Within subject randomized control cross over design	92 male healthy participants	Single dose IN OT (24 IU) or placebo	Overt compliance Lasting changes to memory	OT enhanced compliance with erroneous opinions of others, and decreased influence of others on long term memories
Huang et al. (51)	Randomized double blind placebo controlled trial	85 male participants	Single dose IN OT (24 IU) or placebo	Facial attractiveness judgment scale rating unfamiliar Chinese female faces; subsequently participants were informed of ratings given by peers from an in-group (Chinese) and out-group (Japanese) simultaneously and then were asked to re-rate the same faces	OT increased conformity to both in and out group opinions.
Lane et al. (52)	Two double blind randomized control trials	1st trial-−95 male participants 2nd trial-−61 male participants	Single dose IN OT (32 IU) or placebo	Both trials employed an envelope task exploring trust, compassion and openness	There was no significant effect of in either of the trials
Ruissen et al. (53)	Randomized double blind between subject controlled trial	63 healthy male participants	Single dose IN OT (24 IU) or placebo	Performance individually and on joint completion of the Simon task and EEG recordings during individual and joint performance on the Simon task	There was an enhanced Simon effect in the social context after administration of OT. Oxytocin enhanced self other integration compared with placebo on the N2 component of the EEG
Declereck et al. (54)	Double blind control trial	259 male and female participants	3 doses, at 5 minute intervals of IN OT (24 IU) or placebo	Participants played a range of mixed emotive games (prisoners dilemma); one group was subject to a social cue prior to completion	OT and social cues interact to alter the behaviors of individuals with a pro self-value orientation; after prior contact with the game partner, OT enhances cooperative behavior compared to anonymous conditions where it exacerbates intrinsic self-interest behavior
Shalvi and De Drue (55)	Double blind placebo controlled trial	60 male participants	Single dose IN OT (24 IU) or placebo	Coin toss prediction task; participants were able to report their performance levels dishonestly to benefit their group	Healthy males in OT group lied more to benefit their group and did so faster than placebo group. Treatment effects emerged when lying had financial consequence
Yao et al. (56)	Double blind between subject placebo controlled trial	104 male and female participants	Single dose IN OT (24 IU) or placebo	Revised version of a trust game with 5 players (1 truster, 4 trustees). The participant was always the truster and the trustees were not real	Although OT had no effect on modulating trust restoration, it did have a gender specific effect, with females showing less evidence for trust repair in OT vs. placebo groups
Israel et al. (57)	Randomized placebo control trial	84 male participants	Single dose IN OT (24 IU) or placebo	Clips using an adaptation of the prisoner's dilemma task. Participants' financial awards were contingent on their own and their partner's decisions	OT participants were less accurate than those on placebo at predicting participants' decisions
Rilling et al. (58)	Randomized double blind placebo control trial.	91 male participants	IN OT (24 IU) or Placebo or IN vasopression (140 IU)	Iterative prisoners dilemma game looking at behavioral and fMRI responses.	fMRI results showed that OT, relative to both vasopressin, and placebo increased caudate nucleus response to reciprocated cooperation and left amygdala activation to reciprocated cooperation. Behaviorally OT was associated with increased rates of cooperation
**Studies in aggression and violence**					
Ne'eman et al. (59)	Randomized, double blind, placebo controlled within subject trial	48 healthy adults participants (28 men and 20 women)	IN OT (24 IU) or placebo	Performance on a Social Orientation Paradigm (SOP) to measure for real time aggressive behavior in response to provocation	In those naive to the SOP, oxytocin increased the aggressive response in comparison with placebo
DeWall et al. (60)	Double blind placebo controlled between subject experiment	93 male and female participants	Single dose IN OT (24 IU) or placebo	Participants took part in 2 provocation tasks with participants rating the probability that they would engage in various aggressive behaviors with an intimate partner	OT increased interpersonal violence inclinations but this effect was limited to participants prone to physical aggression
**Studies in moral responsibility**					
Goodyear et al. (61)	Randomized double blind placebo controlled, between subject design	84 male healthy participants	Single dose IN OT (40 IU) or placebo	Intuitions about free will and moral responsibility using ratings of vignettes in deterministic and indeterministic universes	Placebo group held offender more morally responsible compared to OT group
Scheele et al. (62)	Counter balanced, within subject double blind trial	157 male and female participants	Single dose IN OT (24 IU) or placebo	Rating of intensity of own emotional arousal to pictures of faces during fMRI scanning.	OT facilitated cortical midline responses during the self processing of disgust and selectively promoted self interest moral judgments in men. In women OT increased the reaction time difference between accepted and rejected moral dilemmas.

### Study Design

All studies were placebo controlled trials. Twenty five of the included studies were randomized (27, 29–37, 39, 41, 45–53, 57–59, 61). Thirty one studies were double blinded (27, 29–37, 39–42, 44–49, 51–56, 58–62). In addition there were two single blinded studies (28, 43). Three studies were not blinded (38, 50, 57).

### Sample Size

The total number of participants in all of the included studies was 2,615 with study sizes ranging from 6 (28) to 259 participants (54).

### Participants

Fourteen of the studies included male and female participants (27, 29, 33, 37, 40–42, 46, 48, 54, 56, 59, 60, 62). Two studies included only female participants (31, 47). The remaining 20 trials only included male participants (28, 30, 32, 34–36, 38, 39, 43–45, 49–53, 55, 57, 58, 61).

### Oxytocin Administration

All study participants received a single dose of either intranasal (IN) OT or placebo except for one study in which participants were given three doses of intranasal OT at 5-min intervals (54). Doses ranged from 12 to 48 IU per dose.

### Outcome Measures

Included studies used a variety of experiments to assess for different outcomes. These outcome measures included: intuitions about free will and moral responsibility, compliance, memory, social conformity, empathy, facial empathic recognition, inhibitory control, in-group favoritism, aggression, and violence. The experimental paradigms and means of measuring these outcomes varied between studies. These are detailed in Table 1.

## Studies In Participants With ASPD

There were two studies that looked at the effects of OT in participants with ASPD (27, 28).

Timmerman and colleagues conducted a randomized, controlled, double blind, placebo crossover trial. They included 22 adults with ASPD (14 males, 8 females) and 29 healthy controls (11 females, 18 males) in the study. Both before and after IN OT and placebo participants were shown images of faces showing various emotions and assessed on their ability to accurately identify the emotion displayed and the time delay for this. The study found that there were relative deficits in the ASPD group recognizing fearful and happy faces. It was found that following OT administration these effects were no longer observable (27).

Alcorn and colleagues conducted a single blind placebo controlled trial with 6 male participants with ASPD in a community setting. Participants took part in the PSAP (point subtraction aggression paradigm). This is a well-established a validated laboratory measure of state human aggression. Participants were informed that they were anonymously paired with another (fictitious) individual. In their pairs they had a choice to press one of 3 buttons which corresponded to monetary reinforced, aggressive and escape responses. The purpose was for participants to earn as much money as possible. Participants were observed for shifts in their response times on the aggressive response options when having had IN oxytocin or placebo. This study found that there were no specific effect of OT on the aggressive responding. However, there effects were impacted upon by some significant individual differences in responses. There were some individuals who demonstrated a large increases in aggressive responses to the PSAP when given OT but some who demonstrated the opposite effects. The authors concluded that the effects were also not systematically related to dose and that there were no trends between OT and aggressive responses (28).

## Studies In Healthy Participants

There was a great degree of heterogeneity in the studies in healthy participants. There were differences in which aspects of the outcome measures were being assessed and how these were assessed. For convenience we have grouped these studies together under the outcome measures highlighted above.

### Description of Study Findings by Outcome Measure

#### Studies in Empathy

Thirteen studies looked at the effect of OT on empathy (29, 30, 32–42). Empathy was assessed using a variety of tasks.

Hubble and colleagues conducted a randomized, controlled, double blind, placebo within subject trial with 40 healthy males. Participants completed questionnaires which provided empathy scores after being shown video clips that were designed to elicit emotional responses. In addition the eye tracking of the participants was assessed. OT was associated with an increase in time spent fixating upon the eye region of the protagonist's face across emotions. OT also selectively enhanced self-reported affective empathy for fear but not for other emotions. There was no positive relationship between eye gaze patterns and affective empathy (30).

Human and colleagues conducted a randomized, controlled, double blind placebo controlled trial with 116 healthy participants (46 men 70 women). Participants were randomized to receive either IN OT or placebo and completed a series of tasks either with the help from a computer or a confederate human interaction partner. Prior to the main task, the participants undertook a help manipulation task. This was a “tedious” task where participants had to sort letter strings as words or non-words as quickly as possible. During the task, the computer needed fixing and the help manipulation group received input from a technician. Following this help manipulation, the participants undertook two interactive, cooperative tasks. One was a “touch task” (a designed tactile American Sign Language task) which was developed in order to facilitate interpersonal closeness between participants. The second task was a “taboo game” which was similar to an executive functioning tasks that requires response inhibition. The affect and social perception of participants was assessed using a PANAS and participants were asked to rate themselves and their partners. OT administration buffered against the negative subject responses to receiving help that were seen in the placebo group. Those who received oxytocin also expressed greater happiness and gratitude in response to receiving help (29).

Hecht and colleagues conducted a randomized, controlled, double blind placebo controlled trial with 28 healthy female participants. Participants were randomized to receive either 24 IU of intranasal OT or placebo. Participants were then shown animations of geometric shapes depicting social interactions such as playing, chasing, fighting or random movements. Their responses as to whether the shapes represented “friends” or “not friends” were measured, as were their neural responses on fMRI. OT reduced activation in early visual cortex and dorsal-stream motion processing regions. The authors concluded that this indicated that reduced activity was related to social attention. OT also reduced endorsements that shapes were “friends” or “not friends,” and this significantly correlated with reduction in neural activation. Furthermore, participants who perceived fewer social relationships at baseline were more likely to show OT induced increases in a broad network of regions involved in social perception and social cognition, suggesting that lower social processing at baseline may predict more positive neural responses to OT (31).

Li and colleagues conducted a randomized, controlled, double blind, placebo within subject trial with 30 healthy fathers of 1–2 year old children. Participants were randomized to receive IN OT, vasopression, or placebo. Participants were shown photographic stimuli of emotional faces of adults and children. In addition they were exposed to a cry stimulus. Neural responses were examined through fMRI. The study found that OT significantly increased the BOLD fMRI response to viewing pictures of participants' own children in brain regions involved in reward, empathy, and attention in human fathers (32).

Luo and colleagues conducted a randomized, controlled, double blind placebo controlled trial with 86 healthy participants (43 males, 43 females). Participants were randomized to have intranasal OT or placebo and were then shown a range of images of emotional faces. Their brain function was measured with fMRI scans as participants viewed the images. In response to seeing a threatening facial stimuli, in males, OT suppressed the inferior frontal gyrus, dorsal anterior cingulate, and anterior insula responses. In females OT led to an increased response in these areas. The authors concluded that oxytocin produces sex dependent effects in social emotional processing and may have different therapeutic effects on men and women (33).

Strang and colleagues conducted a randomized, controlled, double blind placebo controlled trial with 132 healthy male participants. Following administration of OT or placebo, participants' performance on a task where they could decide how to give of their endowment to a person at a specific social distance. In those who received OT there was a positive correlation between individual trait empathy and the generosity toward others (34).

Hi and colleagues conducted a double blind, randomized cross over trial with 41 healthy male participants. Following administration of OT or placebo, participants took part in a “HelPun” task. In this task participants transfer money from their own endowment to either help a victim or punish a norm violator. Participants' behavior and fMRI scan results were observed. Under OT, participants showed a trend to accelerate altruistic decisions. The enhancement of prosocial-relevant perception was also supported by findings from the fMRI scans, which showed an increase in neural activations in Theory of Mind related neural areas such as the left temporoparietal junction during observations of others being helped (35).

Korb and colleagues conducted a double blind, randomized, placebo controlled, between subject trial with 60 healthy male participants. Following administration of OT or placebo participants were shown a number of stimuli in the form of pictures of expressive faces. The faces gradually changed the expressions between happy, angry, and neutral expressions. Participants were asked to identify when the expression changed. Participants were also asked to rate the intensity of the expression shown. Participants were also instructed to smile or frown in response to instructions on a screen which was assessed using facial EMG. Facial mimicry was increased in the OT group but the effects were strongest in response to angry infant faces. Assessment of the impact of the intensity of the facial expression showed that OT did not modulate facial mimicry in the intensity task (36).

Palgi and colleagues conducted a double blind, within subject placebo randomized controlled trial with 30 male and female participants. Following administration of OT or placebo, participants listened to an audio recording of protagonists of both genders describing distressing emotional conflicts. They were then asked to provide compassionate advice. Two clinical psychologists listened to their recorded responses and then rated their responses for levels of compassion. In both male and female participants OT enhanced compassion toward females but not males (37).

Perry and colleagues conducted a randomized double blind placebo controlled trial with 54 males. Participants were given an online questionnaire which investigated their reactivity to others in order to assess for the participants' global concept of empathy. They then took part in two experiments. The first experiment looked at preferred interpersonal distance with a number of hypothetical protagonists (a friend, a stranger, an authority figure, and a rolling ball). The second experiment involved participants deciding which room they would like to be in depending on different characteristics of the rooms relating to interpersonal distance. The authors found that amongst highly empathetic individuals (as identified by the pre experiment questionnaire) OT promoted a choice of closer interpersonal distances. However, the opposite effect was found with individuals with low empathetic traits. The authors infer from these results that that OT may not have generalized positive effects on individuals with social disorder (38).

Gallup and colleagues conducted a double blind randomized control trial of 60 male healthy participants. Participants were shown a “contagious yawning” video stimulus, and were observed for contagious yawning and other behaviors. Intranasal OT did not increase contagious yawning but modulated expressions that were indicative of awareness of the social stigma associated with this behavior. Those who received OT were more likely to conceal their yawns and were less likely to display overt cues associated with this behavior (39).

Abu-Akel and colleagues conducted a double blind placebo controlled crossover trial of 29 male and female participants. Participants were shown pictures of people with their limbs in various painful situations and were asked to imagine themselves and others in these same painful situations and to give empathetic responses. It was found that OT increased empathy when imagining others compared with imagining oneself in pain; this difference was not found in the placebo group (40).

Cardoso and colleagues conducted a double blind randomized control trial of 82 male and female participants. Participants were asked to complete the perceiving and understanding emotion components of Mayer-Salovey-Caruso Emotional Intelligence Test (MSCEIT). This looked at the effect of oxytocin on perceiving and understanding emotion, on accurate perception of emotions on the Faces Task, and on intensity rating of facial emotions. Participants treated with OT rated the emotion in facial stimuli with greater intensity than those treated with placebo. However, accuracy of emotion identification in faces was impaired in the OT group relative to placebo for all emotions (41).

Fischer-Shofty and colleagues conducted a double blind placebo controlled trial of 62 male and female participants. Participants completed an interpersonal perception task. The authors found that OT improved accuracy of perception of social interactions. In addition OT improved kinship recognition in women but not men. The performance of males was only improved on competition recognition (42).

### Studies in Inhibitory Control

Two studies looked at the effect of OT on inhibitory control (43, 44).

Hirosawa and colleagues conducted a single blind placebo controlled crossover study of 20 male participants. Two paradigms were used: Paradigm 1 investigated the effects of OT on interpretation of facial cognition. Paradigm 2 investigated the effect of OT on attentional-inhibitory control using a modification of the speeded flanker task. OT did not show any effect on either of these tasks. However, the enhancement of attentional-inhibitory control after OT administration significantly correlated with the positively valenced effects of the interpretation of uncertain facial cognition (i.e., neutral and ambiguous facial expressions). That is to say, in those who exhibited a positive beneficial effect of OT on attentional inhibitory control, OT was associated with a tendency to interpret uncertain facial cognitions as being less hostile (43).

Ma and colleagues conducted a double blind placebo controlled between subject experiment with 150 male participants exploring the effects of OT on in-group favoritism where cognitive processing was experimentally manipulated. In addition, individual differences in participants' inclination toward intuition or reflection in daily life were examined. The study's results demonstrated the distinct functional roles of OT when different cognitive styles are promoted during group social cooperation. OT increased in-group favoritism in intuitive participants. However, decreased in-group favoritism was found in those who rely on a reflective style (44).

### Studies in Compliance and Conformity

There were 14 studies that looked at the effect of OT on compliance and conformity; these were assessed through a number of tasks as described below (45–58).

Aygodan and colleagues conducted a randomized double blind placebo controlled trial with 120 healthy male participants. Participants received either intranasal OT or placebo and their performance on a competitive and noncompetitive coin tossing task, where participants had to self-report in order to win a monetary prize, was assessed. This task was to measure conformity to the widely accepted norm of honesty under the pressure of competition in the OT group compared with the placebo group. The study found that conformity was enhanced by OT. In the competitive task OT's positive effect on conformity was associated with a reduction in honesty. In the non-competitive task the opposite was found (45).

Gross and colleagues conducted a randomized double blind placebo controlled within subject trial with 139 healthy participants. Participants received either intranasal OT or placebo and were given a test of conformity to instructions. In the test, participants had a binary choice and were given an arbitrary rule that would mean that they would receive a lesser financial benefit. Under OT participants violated the rule more often. This was most apparent in individuals who had a high need for structure (46).

Lambert and colleagues conducted a randomized double blind placebo controlled trial with 30 healthy females. Participants received either intranasal OT or placebo and their performance on two social dilemma games was measured. At the same time, participants were shown social cues in the form of pictures of neutral or angry faces and also underwent fMRI scanning. The study found that OT significantly increased the activation of the nucleus accumbens during an assurance game that rewards mutual cooperation but significantly attenuated amygdala signal (47).

Ten Velden and colleagues conducted a randomized double blind placebo controlled within subject trial with 65 healthy males and 129 healthy female participants. Participants were placed in groups and given tasks to test the levels of cooperation within the in-group. The task involved the group deciding to make a within group contribution or a between group contribution. Prior to the decision to contribute participants undertook a Stroop Interference task that was either cognitively taxing or not. The study found that participants receiving placebo contributed more to the within group when they were cognitively taxed. The OT group contributed to the within group regardless of cognitive taxation (48).

Hertz and colleagues conducted a randomized double blind placebo controlled trial of 90 healthy male participants. Participants were randomized to receive either placebo or intranasal OT and performed a visual search task in paired dyads. Compared to the placebo group there was a greater collective benefit over time in the OT group. In addition, in the OT group, the more competent member of each dyad was less likely to change their mind during disagreements (49).

Edelson and colleagues conducted a within subject randomized placebo controlled cross over study of 92 male healthy participants. Participants were exposed to erroneous information in various forms as individuals and as a group. Their memory of the events was then assessed in the context of manipulation and no manipulation, with an attempt to induce conformity with peer pressure. It was found that OT enhanced compliance to erroneous opinions of others, and decreased the influence of others' opinions on longer term memories (50).

Huang and colleagues conducted a double blind placebo randomized controlled trial of 85 male participants. They were asked to rate the attractiveness of unfamiliar Chinese faces (from the same ethnicity of the participants); subsequently participants were informed of the ratings of their peers from an ethnic in group (Chinese) and an ethnic out group (Japanese) before being asked to re-rate the initial faces for attractiveness. Results demonstrated that OT promoted conformity regardless of membership of social group when social pressure was applied (51).

Lane and colleagues considered the role of OT administration upon trusting behaviors. This was based upon a previous successful study by Kostfield et al. (63), which demonstrated an increase in trusting behavior with OT. Two double blind randomized controlled trials were conducted with 95 and 61 male participants, respectively. In the first trial participants were given OT or placebo and then asked to complete an “envelope task.” Participants were asked to complete a questionnaire which had questions about the experimenter and intimate questions about the participant. Trust was assessed by the degree of openness of an envelope containing a participant's confidential information. In the second trial participants were given OT or placebo and were then assessed for compassion and openness of responses in a further envelope task. No effects were found on either of these tasks (52).

Ruissen and colleagues conducted a randomized double blind placebo controlled between subject trial of 63 healthy male participants. Following placebo or oxytocin, the performance of participants individually and jointly on completion of the Simon task (a test to investigate modulation of the self-other integration process during joint task performance) were assessed. EEG recordings were also taken. The study found that there was an enhanced Simon effect (positive response to the Simon task measure of self-other integration) in the social context after administration of OT. OT enhanced self-other integration (the ability to integrate of one's own and others actions) compared with placebo. This was apparent on behavioral measures and was also evident in the electrophysiological measures on the EEG (53).

Declereck and colleagues conducted a double blind control trial of 259 male and female participants. Participants played a range of mixed emotive games (prisoner's dilemmas) and one group had a manipulated social cue prior to completion of the task. OT and social cues interacted to alter the behaviors of individuals with a pro self-value orientation. After prior contact with the game partner, OT enhanced cooperative behavior compared to anonymous conditions where it increased intrinsic self-interest behavior (54).

Shalvi and colleagues conducted a double blind placebo controlled trial of 60 male participants. Participants worked in groups and completed a single coin toss prediction task. They were able to dishonestly report their performance levels to benefit their group. Healthy males in the OT group lied more to benefit their group and did so faster than those receiving placebo. These treatment effects were more apparent when lying had financial consequences though lying did not correlate with expected reciprocal dishonesty (55).

Yao and colleagues conducted a double blind between subject placebo controlled trial of 104 male and female participants. Participants took part in a revised version of a trust game with 5 players (1 truster, 4 trustees). The participant was always the truster and the trustees were not real. Although OT had no effect on modulating trust restoration, it did have a gender specific effect, with females showing less evidence of trust repair in the OT vs. the placebo group. The gender specific effect was more evident in the context of attempted trust repair using financial compensation (56).

Israel and colleagues conducted a randomized placebo controlled trial of 84 male participants. Participants were paired and asked to watch clips in an adaptation of the prisoner's dilemma task. Participants' financial awards were contingent on their own and their partner's decisions. People who had been given OT were less accurate than those on placebo at predicting their partner's decisions. The authors concluded that OT appears to impede the accurate assessment of trustworthiness in risky social exchanges (57).

Rilling and colleagues conducted a double blind randomized placebo control trial in 91 male participants. Subjects were given either intranasal OT (24 IU) or intranasal vasopression (140 IU) and both arms had a placebo group. The task used was an iterated prisoners' dilemma game during which the impact of intranasal OT and vasopressin on behavior and brain activity was assessed. fMRI results showed that OT, relative to both vasopressin and placebo, increased responses in the caudate nucleus and left amygdala to reciprocated cooperation. Behaviorally, OT was associated with; increased rates of cooperation, increased facilitation of reward of reciprocated cooperation, increased facilitation of learning that another person can be trusted (58).

### Studies in Aggression and Violence

Two studies looked at the effect of OT on aggression and violence (59, 60).

Ne'eman and colleagues conducted a randomized double blind placebo controlled within subject trial with 28 healthy men and 20 healthy women. Participants were administered OT or placebo before performance on a Social Orientation Paradigm (SOP) to measure for real time aggressive behavior in response to provocation. OT increased the aggressive response in comparison with placebo (59).

DeWall and colleagues conducted a double blind placebo controlled between subject experiment with 93 male and female participants. Participants took part in two provocation tasks rating the probability that they would engage in various aggressive behaviors with an intimate partner. In those given OT there were increased interpersonal violence inclinations but this effect was limited to participants prone to physical aggression in the first place (60).

### Studies in Moral Responsibility

There were two studies that looked at the effect of OT on moral responsibility (61, 62).

Goodyear and colleagues conducted a randomized double blind placebo controlled, between subject study of 84 male healthy participants. Participants were assessed for intuitions about free will and moral responsibility by asking them to rate hypothetical vignettes from deterministic and indeterministic universes. Vignettes related to the moral responsibility of a hypothetical offender. The placebo group held offenders more morally responsible whereas in the OT group participants had greater leniency and assigned less moral responsibility to the offender (61).

Scheele and colleagues conducted a counter balanced, within subject double blind placebo controlled trial of 157 male and female participants. During fMRI scanning, participants rated the intensity of their emotional arousal to a set of pictures of faces. Participants were presented with moral dilemma scenarios and asked how they would respond in these scenarios. It was found that OT facilitated cortical midline responses during the processing of disgust when exposed to pictures of faces. OT was also found to selectively promote self-interested moral judgments in men. In women, OT increased the reaction time in performing on the moral dilemma scenarios (62).

## Discussion

We have conducted a systematic review to examine the effects oxytocin may have in persons with ASPD. After an extensive systematic literature search we found only two studies using oxytocin in participants with ASPD. The lack of research in this area indicates that this is a novel and interesting area that may be the focus of research in the future.

The findings from the studies that have participants with ASPD look specifically at human aggression using the PSAP (28), and the ability to process and interpret emotional faces (27). What we know from these studies is that OT administration in participants with ASPD, corrected the relative deficits in recognizing fearful or happy faces (27). The effect of OT on human aggression, as assessed by the PSAP was found to be not systematically related to dose and there were no trends between OT and aggressive responses. Both of the ASPD studies highlight a number of limitations of their studies, including small sample size and confounding factors such as criminal and drug use histories. Future studies would need to have larger numbers to ensure that they are sufficiently powered in order for the results to be meaningful. It is impressive that in both of the studies there were no drop outs. It would perhaps be expected by the very nature of participants having ASPD, that they may be more likely to drop out. A sufficient number of participants recruited to future studies would help with this. Future studies therefore need to recruit sufficient participant numbers to allow for meaningful control of confounding factors. The ASPD population is a diverse group which can have large numbers of comorbid mental disorders and substance use disorders (2). This is something that would need to be screened for carefully and having such comorbidities could be part of the exclusion criteria for participants. Furthermore, the impact of other complex factors such as criminality and social factors should be accounted for and controlled for to manage the risk of confounding the primary outcome measures. Future studies in participants with ASPD would also need to carefully consider the potential risks associated with a complex ASPD group of participants receiving an intervention. In particular risks of worsening symptoms and causing an increase in the risk related aspects of their presentation.

With a limited number of studies that looked at the use of OT in ASPD we widened our search to include studies that looked at the effects of OT in modulating function in healthy controls that are relevant to the symptomatology of ASPD. We found 34 studies that met our inclusion criteria. All studies were placebo controlled and all but three (38, 50, 57) were randomized and/or double-blinded. This suggests that for most of the included studies there were robust study designs.

The 13 studies that examined the effect of oxytocin on empathy all demonstrated that oxytocin significantly improves empathy. However, the tasks used to assess this were very different. These results show promise for a population with ASPD who inherently have deficits with empathy. However, there are some potential areas of concerns regarding the use of OT in ASPD based on the limited literature described here. For example, one study found that OT worsened the accuracy of interpreting emotions (41). In the ASPD population, which is known to lack empathy as well as impulse control, such an effect would be counterproductive and potentially risky.

There were only two studies that looked at the effect of OT on inhibitory control, an area central to the risks associated with ASPD. Unfortunately, only one of the studies found that OT helped to control inhibition (44). However, even these results highlighted that outcomes were dependent upon individual's baseline traits—highly reflective individuals responded better to OT compared to those with intuitive personality styles. The evidence for using OT in improving inhibitory control is therefore limited. This again highlights the importance of future studies in participants with ASPD controlling for the effects of the individual's baseline traits.

Fourteen studies investigated compliance and conformity out of which seven found improvements in compliance and conformity following OT administration. The tasks used varied between studies and included; competitive and non-competitive coin tossing tasks (45), social dilemma tasks (47), Stroop Interference task (48), tasks with monetary involvement, a visual search task (49), memory under peer pressure (50), judgements of attractiveness between in and out groups (51), envelope task (52), the Simon Task (53), and the prisoners dilemma (54). Whilst it would at first glance seem to hold some promise in managing patients with ASPD a more detailed analysis of the findings raises some concerns. Particularly, in a number of studies compliance and conformity was greater within an “in group.” This would suggest that whilst OT increases compliance it could also increase a person's vulnerability to peer pressure. For patients with ASPD living in institutions with similarly antisocial individuals this would be an undesired effect.

Only one study explored the effect of OT on aggression and violence and results raise further concerns regarding the potential use of OT in this patient group as it appeared to increase inclinations toward aggression and violence in those already prone to violence (60). When compared with the study by Alcorn et al. (28) this raises additional concerns and would indicate a need for future studies to explore this difference in more detail.

The two studies on moral judgement likewise do not show promise; on the contrary OT appears to result in a greater degree of leniency toward offenders, the opposite of a desired effect in ASPD (61). Whilst we are not aware of what the effect it would have in an ASPD cohort, this would be a significant concern and could raise an increase in risks to others. It would indicate that future studies need to manage this risk carefully and assess for the effect of OT on “moral judgement” in the ASPD participants.

Across all the studies one of the challenges is that whilst there are a number of studies in both healthy and ASPD participants which show the effects that OT have there is no consistent evidence that OT has a single and reproducible effect on any one function of human behavior. Some studies looking at the same functions show that OT enhances functions but others looking at the same functions show that OT has the opposite effect. One of the limitations in trying to draw inferences from a wide range of studies is that the populations are heterogenous and this in itself may have a significant impact upon findings and results between studies that investigate the same functions. Our findings are also limited by the absence of studies in the actual target condition, ASPD. Instead we had to rely on proxy evidence using studies investigating the effect of OT on relevant functions in healthy individuals. It is not possible to know, on the basis of the available evidence to date, whether findings from healthy groups can be extrapolated to personality disordered individuals. Even within healthy individuals, in each of the symptom groups there was little uniformity between studies in terms of the tasks or outcome measures used. This is perhaps not surprising as the symptoms groups are complex to define and assess. Furthermore, whilst the studies only included healthy adults, in the absence of personality assessments in the included participants, one cannot rule out the possibility of confounding effects in the findings.

## Conclusion

This is the first systematic literature review exploring the potential use of oxytocin in managing the symptoms of ASPD. It is apparent that there is a reasonable body of evidence addressing related symptoms in healthy individuals, but only two studies including participants with ASPD. The majority of studies were large sample, randomized controlled trials exploring a range of functions, including interpersonal relationships, compliance, empathy, emotional processing, moral judgment, deceitfulness, and conformity. Findings were highly dependent upon context and the participants' premorbid states. OT has been shown to demonstrate diversified effects, in most cases being associated with socially positive or non-criminogenic behaviors. However, some studies found opposite, and non-desirable, effects, e.g., an increase in violent inclinations. It is also of note that ASPD symptoms do not occur in isolation and there is likely to be a complex interplay between symptoms. It is difficult therefore to draw any direct inferences from healthy control studies. Further high quality large sample studies are required to explore the benefits of oxytocin in a population with an established diagnosis of ASPD. Studies should also rigorously control for potential confounding effects.

## Author Contributions

All authors contributed to this paper including the planning, development of a search strategy, reviewing the papers, and writing the article.

### Conflict of Interest Statement

The authors declare that the research was conducted in the absence of any commercial or financial relationships that could be construed as a potential conflict of interest.
