# Supplementary material for: The Role of Oxytocin in Antisocial Personality Disorders: A Systematic Review of the Literature
Source: Front Psychiatry. 2019 Feb 27;10:76. doi: 10.3389/fpsyt.2019.00076 (PMC6400857; doi:10.3389/fpsyt.2019.00076)
Supplement: Supplementary file 1 [file Data_Sheet_1.docx]

**The role of Oxytocin in Antisocial Personality Disorders: A systematic review of the literature**

**Appendix 1: Search Terms and Databases**

**Search History – Oxytocin and ASPD**:

|  | Database | Search Term |
| --- | --- | --- |
| 1 | PsycInfo | OXYTOCIN/ |
| 2 | PsycInfo | oxytocin.af |
| 3 | PsycInfo | 1 OR 2 |
| 4 | PsycInfo | ANTISOCIAL PERSONALITY DISORDER/ |
| 5 | PsycInfo | ASPD.af |
| 6 | PsycInfo | "Antisocial personalit*".af |
| 7 | PsycInfo | "anti social".af |
| 8 | PsycInfo | "asocial".af |
| 9 | PsycInfo | Psychopath*.af |
| 10 | PsycInfo | sociopath*.af |
| 11 | PsycInfo | Dyssocial.af |
| 12 | PsycInfo | "dissocial personalit*".af |
| 13 | PsycInfo | "moral insanity".af |
| 14 | PsycInfo | "Dangerous and Severe Personality Disorder".af |
| 15 | PsycInfo | DSPD.af |
| 16 | PsycInfo | SADISM/ |
| 17 | PsycInfo | sadism.af |
| 18 | PsycInfo | sadist*.af |
| 19 | PsycInfo | 4 OR 5 OR 6 OR 7 OR 8 OR 9 OR 10 OR 11 OR 12 OR 13 OR 14 OR 15 OR 16 OR 17 OR 18 |
| 20 | PsycInfo | 3 AND 19 |
| 21 | PsycInfo | 20 [Limit to: (Population Human)] |
| 22 | EMBASE | OXYTOCIN/ |
| 23 | EMBASE | oxytocin.af |
| 24 | EMBASE | 22 OR 23 |
| 25 | EMBASE | ANTISOCIAL PERSONALITY/ OR ANTISOCIAL PERSONALITY DISORDER/ |
| 26 | EMBASE | ASPD.af |
| 27 | EMBASE | "Antisocial personalit*".af |
| 28 | EMBASE | "anti social".af |
| 29 | EMBASE | "asocial".af |
| 30 | EMBASE | PSYCHOPATHY/ |
| 31 | EMBASE | Psychopath*.af |
| 32 | EMBASE | SOCIOPATHY/ |
| 33 | EMBASE | sociopath*.af |
| 34 | EMBASE | Dyssocial.af |
| 35 | EMBASE | "dissocial personalit*".af |
| 36 | EMBASE | "moral insanity".af |
| 37 | EMBASE | "Dangerous and Severe Personality Disorder".af |
| 38 | EMBASE | DSPD.af |
| 39 | EMBASE | SADISM/ |
| 40 | EMBASE | sadism.af |
| 41 | EMBASE | sadist*.af |
| 42 | EMBASE | 25 OR 26 OR 27 OR 28 OR 29 OR 30 OR 31 OR 32 OR 33 OR 34 OR 35 OR 36 OR 37 OR 38 OR 39 OR 40 OR 41 |
| 43 | EMBASE | 24 AND 42 |
| 44 | EMBASE | 43 [Limit to: Human] |
| 45 | Medline | OXYTOCIN/ |
| 46 | Medline | oxytocin.af |
| 47 | Medline | 45 OR 46 |
| 48 | Medline | ANTISOCIAL PERSONALITY DISORDER/ |
| 49 | Medline | ASPD.af |
| 50 | Medline | "Antisocial personalit*".af |
| 51 | Medline | "anti social".af |
| 52 | Medline | "asocial".af |
| 53 | Medline | PSYCHOPATHOLOGY/ |
| 54 | Medline | Psychopath*.af |
| 55 | Medline | sociopath*.af |
| 56 | Medline | Dyssocial.af |
| 57 | Medline | "dissocial personalit*".af |
| 58 | Medline | "moral insanity".af |
| 59 | Medline | "Dangerous and Severe Personality Disorder".af |
| 60 | Medline | DSPD.af |
| 61 | Medline | SADISM/ |
| 62 | Medline | sadism.af |
| 63 | Medline | sadist*.af |
| 64 | Medline | 48 OR 49 OR 50 OR 51 OR 52 OR 53 OR 54 OR 55 OR 56 OR 57 OR 58 OR 59 OR 60 OR 61 OR 62 OR 63 |
| 65 | Medline | 47 AND 64 |
| 66 | Medline | 65 [Limit to: Humans] |
| 67 | CINAHL | OXYTOCICS/ |
| 68 | CINAHL | oxytocin.af |
| 69 | CINAHL | 67 OR 68 |
| 70 | CINAHL | ANTISOCIAL PERSONALITY DISORDER/ |
| 71 | CINAHL | ASPD.af |
| 72 | CINAHL | "Antisocial personalit*".af |
| 73 | CINAHL | "anti social".af |
| 74 | CINAHL | "asocial".af |
| 75 | CINAHL | Psychopath*.af |
| 76 | CINAHL | sociopath*.af |
| 77 | CINAHL | Dyssocial.af |
| 78 | CINAHL | "dissocial personalit*".af |
| 79 | CINAHL | "moral insanity".af |
| 80 | CINAHL | "Dangerous and Severe Personality Disorder".af |
| 81 | CINAHL | DSPD.af |
| 82 | CINAHL | sadism.af |
| 83 | CINAHL | sadist*.af |
| 84 | CINAHL | 70 OR 71 OR 72 OR 73 OR 74 OR 75 OR 76 OR 77 OR 78 OR 79 OR 80 OR 81 OR 82 OR 83 |
| 85 | CINAHL | 69 AND 84 |

Cochrane library

#1 MeSH descriptor: [Oxytocin] this term only

#2 oxytocin:ti,ab,kw (Word variations have been searched)

#3 #1 or #2

#4 MeSH descriptor: [Antisocial Personality Disorder] this term only

#5 ASPD:ti,ab,kw (Word variations have been searched)

#6 "Antisocial personalit*":ti,ab,kw (Word variations have been searched)

#7 "anti social":ti,ab,kw (Word variations have been searched)

#8 "asocial":ti,ab,kw (Word variations have been searched)

#9 MeSH descriptor: [Psychopathology] this term only

#10 Psychopath*:ti,ab,kw (Word variations have been searched)

#11 sociopath*:ti,ab,kw (Word variations have been searched)

#12 Dyssocial:ti,ab,kw (Word variations have been searched)

#13 "dissocial personalit*":ti,ab,kw (Word variations have been searched)

#14 "moral insanity":ti,ab,kw (Word variations have been searched)

#15 "Dangerous and Severe Personality Disorder":ti,ab,kw (Word variations have been searched)

#16 DSPD:ti,ab,kw (Word variations have been searched)

#17 MeSH descriptor: [Sadism] this term only

#18 sadism:ti,ab,kw (Word variations have been searched)

#19 sadist*:ti,ab,kw (Word variations have been searched)

#20 #4 or #5 or #6 or #7 or #8 or #9 or #10 or #11 or #12 or #13 or #14 or #15 or #16 or #17 or #18 or #19

#21 #3 and #20

Applied Social Sciences Index and Abstracts (ASSIA)

Set#: S1

Searched for: OXYTOCIN

Databases: Applied Social Sciences Index and Abstracts (ASSIA)

Set#: S3

Searched for: (Antisocial personalit*) OR (anti social OR asocial) OR (Psychopath* OR sociopath*) OR (Dyssocial OR dissocial personalit*) OR (moral insanity OR dangerous and severe personality disorder) OR (DSPD OR sadism) OR sadist

Databases: Applied Social Sciences Index and Abstracts (ASSIA)

Set#: S4

Searched for: OXYTOCIN AND ((Antisocial personalit*) OR (anti social OR asocial) OR (Psychopath* OR sociopath*) OR (Dyssocial OR dissocial personalit*) OR (moral insanity OR dangerous and severe personality disorder) OR (DSPD OR sadism) OR sadist)

Databases: Applied Social Sciences Index and Abstracts (ASSIA)

Sociological Abstracts

Set#: S9

Searched for: ALL (OXYTOCIN)

Databases: Sociological Abstracts

Set#: S10

Searched for: ALL (Antisocial personalit* OR anti social OR asocial OR Psychopath* OR sociopath* OR Dyssocial OR dissocial personalit* OR moral insanity OR dangerous and severe personality disorder OR DSPD OR sadism OR sadist)

Databases: Sociological Abstracts

Set#: S11

Searched for: (ALL (OXYTOCIN)) AND (ALL (Antisocial personalit* OR anti social OR asocial OR Psychopath* OR sociopath* OR Dyssocial OR dissocial personalit* OR moral insanity OR dangerous and severe personality disorder OR DSPD OR sadism OR sadist))

Databases: Sociological Abstracts

BIOSIS Citation Index

**TITLE:** (OXYTOCIN) *AND* **TITLE:** ("Antisocial personalit*" OR "anti social" OR "asocial" OR Psychopath* OR sociopath* OR Dyssocial OR "dissocial personalit*" OR "moral insanity" OR "dangerous and severe personality disorder" OR DSPD OR sadism OR sadist)

Web of Science

**TITLE:** (OXYTOCIN) *AND* **TITLE:** ("Antisocial personalit*" OR "anti social" OR "asocial" OR Psychopath* OR sociopath* OR Dyssocial OR "dissocial personalit*" OR "moral insanity" OR "dangerous and severe personality disorder" OR DSPD OR sadism OR sadist)

**Search History – Oxytocin and terms associated with ASPD**:

|  | Database | Search Term |
| --- | --- | --- |
| 1 | PsycINFO | OXYTOCIN/ |
| 2 | PsycINFO | (oxytocin).ti,ab |
| 3 | PsycINFO | (1 OR 2) |
| 4 | PsycINFO | "NONCONFORMITY (PERSONALITY)"/ |
| 5 | PsycINFO | ("non compliant" OR Comply OR Conform* OR Disobey OR Follow OR Obedience OR Obey OR Oppose OR Rebel* OR Refuse OR Rules).ti,ab |
| 6 | PsycINFO | EMPATHY/ |
| 7 | PsycINFO | REGRET/ |
| 8 | PsycINFO | SHAME/ |
| 9 | PsycINFO | SADNESS/ |
| 10 | PsycINFO | GRIEF/ |
| 11 | PsycINFO | CRUELTY/ |
| 12 | PsycINFO | GUILT/ |
| 13 | PsycINFO | (Apathetic OR Callous* OR Compassion OR Cruel* OR Empathy OR Grief OR Guilt OR Insensitive OR Penance OR Regret OR Remorse OR Repent* OR Sadness OR Shame OR Sympathy OR Uncaring).ti,ab |
| 14 | PsycINFO | exp VIOLENCE/ |
| 15 | PsycINFO | CRIMINAL BEHAVIOR/ |
| 16 | PsycINFO | CRIME/ |
| 17 | PsycINFO | exp DECEPTION/ |
| 18 | PsycINFO | ("impulse control" OR Aggress* OR Careless OR Conning OR Crime OR Criminal* OR Deceit OR Deceive OR Deception OR Defraud OR Immoral OR Impetuous OR Impulsiv* OR Irresponsib* OR Lie OR Lying OR Manipulation OR Offend* OR Reckless OR Thoughtless OR Violen*).ti,ab |
| 19 | PsycINFO | (4 OR 5 OR 6 OR 7 OR 8 OR 9 OR 10 OR 11 OR 12 OR 13 OR 14 OR 15 OR 16 OR 17 OR 18) |
| 20 | PsycINFO | (3 AND 19) |
| 21 | PsycINFO | 20 [Population Human] |
| 22 | EMBASE | OXYTOCIN/ |
| 23 | EMBASE | (oxytocin).ti,ab |
| 24 | EMBASE | (22 OR 23) |
| 25 | EMBASE | ("non compliant" OR Comply OR Conform* OR Disobey OR Follow OR Obedience OR Obey OR Oppose OR Rebel* OR Refuse OR Rules).ti,ab |
| 26 | EMBASE | EMPATHY/ |
| 27 | EMBASE | SHAME/ |
| 28 | EMBASE | SADNESS/ |
| 29 | EMBASE | GRIEF/ |
| 30 | EMBASE | GUILT/ |
| 31 | EMBASE | (Apathetic OR Callous* OR Compassion OR Cruel* OR Empathy OR Grief OR Guilt OR Insensitive OR Penance OR Regret OR Remorse OR Repent* OR Sadness OR Shame OR Sympathy OR Uncaring).ti,ab |
| 32 | EMBASE | exp VIOLENCE/ |
| 33 | EMBASE | CRIMINAL BEHAVIOR/ |
| 34 | EMBASE | CRIME/ |
| 35 | EMBASE | exp DECEPTION/ |
| 36 | EMBASE | FRAUD/ |
| 37 | EMBASE | IMPULSIVENESS/ |
| 38 | EMBASE | ("impulse control" OR Aggress* OR Careless OR Conning OR Crime OR Criminal* OR Deceit OR Deceive OR Deception OR Defraud OR Immoral OR Impetuous OR Impulsiv* OR Irresponsib* OR Lie OR Lying OR Manipulation OR Offend* OR Reckless OR Thoughtless OR Violen*).ti,ab |
| 39 | EMBASE | (25 OR 26 OR 27 OR 28 OR 29 OR 30 OR 31 OR 32 OR 33 OR 34 OR 35 OR 36 OR 37 OR 38) |
| 40 | EMBASE | (24 AND 39) |
| 41 | EMBASE | 40 [Humans] |
| 42 | Medline | OXYTOCIN/ |
| 43 | Medline | (oxytocin).ti,ab |
| 44 | Medline | (42 OR 43) |
| 45 | Medline | ("non compliant" OR Comply OR Conform* OR Disobey OR Follow OR Obedience OR Obey OR Oppose OR Rebel* OR Refuse OR Rules).ti,ab |
| 46 | Medline | EMPATHY/ |
| 47 | Medline | SHAME/ |
| 48 | Medline | GRIEF/ |
| 49 | Medline | GUILT/ |
| 50 | Medline | (Apathetic OR Callous* OR Compassion OR Cruel* OR Empathy OR Grief OR Guilt OR Insensitive OR Penance OR Regret OR Remorse OR Repent* OR Sadness OR Shame OR Sympathy OR Uncaring).ti,ab |
| 51 | Medline | exp VIOLENCE/ |
| 52 | Medline | CRIME/ |
| 53 | Medline | DECEPTION/ |
| 54 | Medline | FRAUD/ |
| 55 | Medline | IMPULSIVE BEHAVIOR/ |
| 56 | Medline | ("impulse control" OR Aggress* OR Careless OR Conning OR Crime OR Criminal* OR Deceit OR Deceive OR Deception OR Defraud OR Immoral OR Impetuous OR Impulsiv* OR Irresponsib* OR Lie OR Lying OR Manipulation OR Offend* OR Reckless OR Thoughtless OR Violen*).ti,ab |
| 57 | Medline | (45 OR 46 OR 47 OR 48 OR 49 OR 50 OR 51 OR 52 OR 53 OR 54 OR 55 OR 56) |
| 58 | Medline | (44 AND 57) |
| 59 | Medline | 58 [Humans] |
| 60 | CINAHL | OXYTOCIN/ |
| 61 | CINAHL | (oxytocin).ti,ab |
| 62 | CINAHL | (60 OR 61) |
| 63 | CINAHL | ("non compliant" OR Comply OR Conform* OR Disobey OR Follow OR Obedience OR Obey OR Oppose OR Rebel* OR Refuse OR Rules).ti,ab |
| 64 | CINAHL | EMPATHY/ |
| 65 | CINAHL | SHAME/ |
| 66 | CINAHL | GRIEF/ |
| 67 | CINAHL | GUILT/ |
| 68 | CINAHL | (Apathetic OR Callous* OR Compassion OR Cruel* OR Empathy OR Grief OR Guilt OR Insensitive OR Penance OR Regret OR Remorse OR Repent* OR Sadness OR Shame OR Sympathy OR Uncaring).ti,ab |
| 69 | CINAHL | exp VIOLENCE/ |
| 70 | CINAHL | CRIME/ |
| 71 | CINAHL | exp DECEPTION/ |
| 72 | CINAHL | FRAUD/ |
| 73 | CINAHL | ("impulse control" OR Aggress* OR Careless OR Conning OR Crime OR Criminal* OR Deceit OR Deceive OR Deception OR Defraud OR Immoral OR Impetuous OR Impulsiv* OR Irresponsib* OR Lie OR Lying OR Manipulation OR Offend* OR Reckless OR Thoughtless OR Violen*).ti,ab |
| 74 | CINAHL | (63 OR 64 OR 65 OR 66 OR 67 OR 68 OR 69 OR 70 OR 71 OR 72 OR 73) |
| 75 | CINAHL | (62 AND 74) |

Cochrane Library

#1 MeSH descriptor: [Oxytocin] this term only

#2 oxytocin:ti,ab,kw (Word variations have been searched)

#3 #1 or #2

#4 ("non compliant" or Comply or Conform* or Disobey or Follow or Obedience or Obey or Oppose or Rebel* or Refuse or Rules):ti,ab,kw (Word variations have been searched)

#5 MeSH descriptor: [Empathy] this term only

#6 MeSH descriptor: [Guilt] this term only

#7 MeSH descriptor: [Shame] this term only

#8 MeSH descriptor: [Grief] this term only

#9 (Apathetic or Callous* or Compassion or Cruel* or Empathy or Grief or Guilt or Insensitive or Penance or Regret or Remorse or Repent* or Sadness or Shame or Sympathy or Uncaring):ti,ab,kw (Word variations have been searched)

#10 MeSH descriptor: [Violence] explode all trees

#11 MeSH descriptor: [Crime] this term only

#12 MeSH descriptor: [Deception] this term only

#13 MeSH descriptor: [Fraud] this term only

#14 MeSH descriptor: [Impulsive Behavior] this term only

#15 ("impulse control" or Aggress* or Careless or Conning or Crime or Criminal* or Deceit or Deceive or Deception or Defraud or Immoral or Impetuous or Impulsiv* or Irresponsib* or Lie or Lying or Manipulation or Offend* or Reckless or Thoughtless or Violen*):ti,ab,kw (Word variations have been searched)

#16 #4 or #5 or #6 or #7 or #8 or #9 or #10 or #11 or #12 or #13 or #14 or #15

#17 #3 and #16

Applied Social Sciences Index and Abstracts (ASSIA)

su(Oxytocin AND ("non compliant" OR Comply OR Conform* OR Disobey OR Follow OR Obedience OR Obey OR Oppose OR Rebel* OR Refuse OR Rules OR Apathetic OR Callous* OR Compassion OR Cruel* OR Empathy OR Grief OR Guilt OR Insensitive OR Penance OR Regret OR Remorse OR Repent* OR Sadness OR Shame OR Sympathy OR Uncaring OR Apathetic OR Callous* OR Compassion OR Cruel* OR Empathy OR Grief OR Guilt OR Insensitive OR Penance OR Regret OR Remorse OR Repent* OR Sadness OR Shame OR Sympathy OR Uncaring)) OR ti(Oxytocin AND ("non compliant" OR Comply OR Conform* OR Disobey OR Follow OR Obedience OR Obey OR Oppose OR Rebel* OR Refuse OR Rules OR Apathetic OR Callous* OR Compassion OR Cruel* OR Empathy OR Grief OR Guilt OR Insensitive OR Penance OR Regret OR Remorse OR Repent* OR Sadness OR Shame OR Sympathy OR Uncaring OR Apathetic OR Callous* OR Compassion OR Cruel* OR Empathy OR Grief OR Guilt OR Insensitive OR Penance OR Regret OR Remorse OR Repent* OR Sadness OR Shame OR Sympathy OR Uncaring)) OR ab(Oxytocin AND ("non compliant" OR Comply OR Conform* OR Disobey OR Follow OR Obedience OR Obey OR Oppose OR Rebel* OR Refuse OR Rules OR Apathetic OR Callous* OR Compassion OR Cruel* OR Empathy OR Grief OR Guilt OR Insensitive OR Penance OR Regret OR Remorse OR Repent* OR Sadness OR Shame OR Sympathy OR Uncaring OR Apathetic OR Callous* OR Compassion OR Cruel* OR Empathy OR Grief OR Guilt OR Insensitive OR Penance OR Regret OR Remorse OR Repent* OR Sadness OR Shame OR Sympathy OR Uncaring))

Sociological Abstracts

su(Oxytocin AND ("non compliant" OR Comply OR Conform* OR Disobey OR Follow OR Obedience OR Obey OR Oppose OR Rebel* OR Refuse OR Rules or Apathetic OR Callous* OR Compassion OR Cruel* OR Empathy OR Grief OR Guilt OR Insensitive OR Penance OR Regret OR Remorse OR Repent* OR Sadness OR Shame OR Sympathy OR Uncaring or Apathetic OR Callous* OR Compassion OR Cruel* OR Empathy OR Grief OR Guilt OR Insensitive OR Penance OR Regret OR Remorse OR Repent* OR Sadness OR Shame OR Sympathy OR Uncaring)) OR ti(Oxytocin AND ("non compliant" OR Comply OR Conform* OR Disobey OR Follow OR Obedience OR Obey OR Oppose OR Rebel* OR Refuse OR Rules or Apathetic OR Callous* OR Compassion OR Cruel* OR Empathy OR Grief OR Guilt OR Insensitive OR Penance OR Regret OR Remorse OR Repent* OR Sadness OR Shame OR Sympathy OR Uncaring or Apathetic OR Callous* OR Compassion OR Cruel* OR Empathy OR Grief OR Guilt OR Insensitive OR Penance OR Regret OR Remorse OR Repent* OR Sadness OR Shame OR Sympathy OR Uncaring)) OR ab(Oxytocin AND ("non compliant" OR Comply OR Conform* OR Disobey OR Follow OR Obedience OR Obey OR Oppose OR Rebel* OR Refuse OR Rules or Apathetic OR Callous* OR Compassion OR Cruel* OR Empathy OR Grief OR Guilt OR Insensitive OR Penance OR Regret OR Remorse OR Repent* OR Sadness OR Shame OR Sympathy OR Uncaring or Apathetic OR Callous* OR Compassion OR Cruel* OR Empathy OR Grief OR Guilt OR Insensitive OR Penance OR Regret OR Remorse OR Repent* OR Sadness OR Shame OR Sympathy OR Uncaring))

BIOSIS Citation Index

**TITLE:** (Oxytocin) *AND* **TITLE:** (("non compliant" OR Comply OR Conform* OR Disobey OR Follow OR Obedience OR Obey OR Oppose OR Rebel* OR Refuse OR Rules or Apathetic OR Callous* OR Compassion OR Cruel* OR Empathy OR Grief OR Guilt OR Insensitive OR Penance OR Regret OR Remorse OR Repent* OR Sadness OR Shame OR Sympathy OR Uncaring or Apathetic OR Callous* OR Compassion OR Cruel* OR Empathy OR Grief OR Guilt OR Insensitive OR Penance OR Regret OR Remorse OR Repent* OR Sadness OR Shame OR Sympathy OR Uncaring))

Web of science

**TITLE:** (Oxytocin AND ("non compliant" OR Comply OR Conform* OR Disobey OR Follow OR Obedience OR Obey OR Oppose OR Rebel* OR Refuse OR Rules or Apathetic OR Callous* OR Compassion OR Cruel* OR Empathy OR Grief OR Guilt OR Insensitive OR Penance OR Regret OR Remorse OR Repent* OR Sadness OR Shame OR Sympathy OR Uncaring or Apathetic OR Callous* OR Compassion OR Cruel* OR Empathy OR Grief OR Guilt OR Insensitive OR Penance OR Regret OR Remorse OR Repent* OR Sadness OR Shame OR Sympathy OR Uncaring)) *NOT* **TITLE:**(rat or rats or mouse or mice or murine or pig* or swine* or dog* or cat* or feline or rabbit* or primate or monke* or animal* or "in vivo" or model* or experiment*)

*Indexes=SCI-EXPANDED, SSCI, A&HCI, CPCI-S, CPCI-SSH, BKCI-S, BKCI-SSH, CCR-EXPANDED, IC*

Cochrane library

#1 MeSH descriptor: [Oxytocin] this term only

#2 oxytocin:ti,ab,kw (Word variations have been searched)

#3 #1 or #2

#4 MeSH descriptor: [Antisocial Personality Disorder] this term only

#5 ASPD:ti,ab,kw (Word variations have been searched)

#6 "Antisocial personalit*":ti,ab,kw (Word variations have been searched)

#7 "anti social":ti,ab,kw (Word variations have been searched)

#8 "asocial":ti,ab,kw (Word variations have been searched)

#9 MeSH descriptor: [Psychopathology] this term only

#10 Psychopath*:ti,ab,kw (Word variations have been searched)

#11 sociopath*:ti,ab,kw (Word variations have been searched)

#12 Dyssocial:ti,ab,kw (Word variations have been searched)

#13 "dissocial personalit*":ti,ab,kw (Word variations have been searched)

#14 "moral insanity":ti,ab,kw (Word variations have been searched)

#15 "Dangerous and Severe Personality Disorder":ti,ab,kw (Word variations have been searched)

#16 DSPD:ti,ab,kw (Word variations have been searched)

#17 MeSH descriptor: [Sadism] this term only

#18 sadism:ti,ab,kw (Word variations have been searched)

#19 sadist*:ti,ab,kw (Word variations have been searched)

#20 #4 or #5 or #6 or #7 or #8 or #9 or #10 or #11 or #12 or #13 or #14 or #15 or #16 or #17 or #18 or #19

#21 #3 and #20

Applied Social Sciences Index and Abstracts (ASSIA)

Set#: S1

Searched for: OXYTOCIN

Databases: Applied Social Sciences Index and Abstracts (ASSIA)

Set#: S3

Searched for: (Antisocial personalit*) OR (anti social OR asocial) OR (Psychopath* OR sociopath*) OR (Dyssocial OR dissocial personalit*) OR (moral insanity OR dangerous and severe personality disorder) OR (DSPD OR sadism) OR sadist

Databases: Applied Social Sciences Index and Abstracts (ASSIA)

Set#: S4

Searched for: OXYTOCIN AND ((Antisocial personalit*) OR (anti social OR asocial) OR (Psychopath* OR sociopath*) OR (Dyssocial OR dissocial personalit*) OR (moral insanity OR dangerous and severe personality disorder) OR (DSPD OR sadism) OR sadist)

Databases: Applied Social Sciences Index and Abstracts (ASSIA)

Sociological Abstracts

Set#: S9

Searched for: ALL (OXYTOCIN)

Databases: Sociological Abstracts

Set#: S10

Searched for: ALL (Antisocial personalit* OR anti social OR asocial OR Psychopath* OR sociopath* OR Dyssocial OR dissocial personalit* OR moral insanity OR dangerous and severe personality disorder OR DSPD OR sadism OR sadist)

Databases: Sociological Abstracts

Set#: S11

Searched for: (ALL (OXYTOCIN)) AND (ALL (Antisocial personalit* OR anti social OR asocial OR Psychopath* OR sociopath* OR Dyssocial OR dissocial personalit* OR moral insanity OR dangerous and severe personality disorder OR DSPD OR sadism OR sadist))

Databases: Sociological Abstracts

BIOSIS Citation Index

**TITLE:** (OXYTOCIN) *AND* **TITLE:** ("Antisocial personalit*" OR "anti social" OR "asocial" OR Psychopath* OR sociopath* OR Dyssocial OR "dissocial personalit*" OR "moral insanity" OR "dangerous and severe personality disorder" OR DSPD OR sadism OR sadist)

Web of Science

**TITLE:** (OXYTOCIN) *AND* **TITLE:** ("Antisocial personalit*" OR "anti social" OR "asocial" OR Psychopath* OR sociopath* OR Dyssocial OR "dissocial personalit*" OR "moral insanity" OR "dangerous and severe personality disorder" OR DSPD OR sadism OR sadist)
